# Retention of mothers and infants in the prevention of mother-to-child transmission of HIV programme is associated with individual and facility-level factors in Rwanda

**DOI:** 10.7448/IAS.19.5.20837

**Published:** 2016-07-20

**Authors:** Godfrey B Woelk, Dieudonne Ndatimana, Sally Behan, Martha Mukaminega, Epiphanie Nyirabahizi, Heather J Hoffman, Placidie Mugwaneza, Muhayimpundu Ribakare, Anouk Amzel, B Ryan Phelps

**Affiliations:** 1 Elizabeth Glaser Pediatric AIDS Foundation, Washington, DC, USA; 2 Elizabeth Glaser Pediatric AIDS Foundation, Kigali, Rwanda; 3 Department of Epidemiology and Biostatistics, Milken Institute School of Public Health, George Washington University, Washington, DC, USA; 4 Rwanda Biomedical Center, Ministry of Health, Kigali, Rwanda; 5 Office of HIV/AIDS, United States Agency for International Development, Washington, DC, USA

**Keywords:** PMTCT, retention, health facilities, sub-Sahara Africa, Rwanda, mother-infant pairs

## Abstract

**Objectives:**

Investigate levels of retention at specified time periods along the prevention of mother-to-child transmission (PMTCT) cascade among mother-infant pairs as well as individual- and facility-level factors associated with retention.

**Methods:**

A retrospective cohort of HIV-positive pregnant women and their infants attending five health centres from November 2010 to February 2012 in the Option B programme in Rwanda was established. Data were collected from several health registers and patient follow-up files. Additionally, informant interviews were conducted to ascertain health facility characteristics. Generalized estimating equation methods and modelling were utilized to estimate the number of mothers attending each antenatal care visit and assess factors associated with retention.

**Results:**

Data from 457 pregnant women and 462 infants were collected at five different health centres (three urban and two rural facilities). Retention at 30 days after registration and retention at 6 weeks, 3, 6, 9 and 12 months post-delivery were analyzed. Based on an analytical sample of 348, we found that 58% of women and 81% of infants were retained in care within the same health facility at 12 months post-delivery, respectively. However, for mother-infant paired mothers, retention at 12 months was 74% and 79% for their infants. Loss to facility occurred early, with 26% to 33% being lost within 30 days post-registration. In a multivariable model retention was associated with being married, adjusted relative risk (ARR): 1.26, (95% confidence intervals: 1.11, 1.43); antiretroviral therapy eligible, ARR: 1.39, (1.12, 1.73) and CD4 count per 50 mm^3^, ARR: 1.02, (1.01, 1.03).

**Conclusions:**

These findings demonstrate varying retention levels among mother-infant pairs along the PMTCT cascade in addition to potential determinants of retention to such programmes. Unmarried, apparently healthy, HIV-positive pregnant women need additional support for programme retention. With the significantly increased workload resulting from lifelong antiretroviral treatment for all HIV-positive pregnant women, strategies need to be developed to identify, provide support and trace these women at risk of loss to follow-up. This study provides further evidence for the need for such a targeted supportive approach.

## Introduction

There has been significant progress in the prevention of mother-to-child transmission (PMTCT) of HIV globally. The number of new infections among children decreased by 58% between 2000 and 2014; from 520,000 to 220,000 [1]. The continued success of efforts to combat mother-to-child transmission supports our ultimate goal of being able to eliminate new infections among children globally [2]. This ambitious goal appears feasible if existing resources are used wisely and obstacles are anticipated and overcome. In particular, the PMTCT programme retention of mothers during pregnancy and mothers and infants post-delivery will be vital to achieving this goal. Women and infants who are retained in care have better health outcomes, and women who are retained and adhere to their antiretroviral therapy (ART) are less likely to transmit HIV to others [3,4]. However, HIV-positive pregnant women are less likely to be retained in care than HIV-positive non-pregnant women and men [5–7].

There are several barriers to retention among women and infants in PMTCT programmes. Data suggest that transportation costs, stigma and discrimination and fear of HIV status disclosure, comprise some of the major impediments to an individual continuing in care [8–12]. It is important to understand both individual and facility factors affecting retention along the PMTCT cascade.

Health facility characteristics that may influence retention include both the structural and operational aspects of a facility such as capacity, location, staffing and services provided. One cross-sectional study among HIV-positive post-partum women in Ethiopia found that the likelihood of receiving PMTCT services increased by 7.2 times with every additional nurse per 1500 patients, indicating patient load may be associated with retention [13]. Another study in South Africa found that patients receiving HIV treatment at local clinics were less likely to be lost to follow-up than patients treated at hospitals [14]. Further, a retrospective cohort study conducted in Malawi among women enrolled in Option B+ programmes found a positive, though weak, association between rural facility location and retention as well as a negative association between attending larger facilities and retention [15].

Overall, these individual- and facility-level barriers result in decreased retention among women and infants throughout the PMTCT cascade [16,17], with only 66% of HIV-positive pregnant women receiving ART and 49% of infants receiving virologic diagnostic testing within the first two months of life in 2014 [18]. Understanding how these barriers impact retention among women and infants at varying time intervals along the PMTCT cascade may contribute to optimizing the performance of PMTCT programmes and identifying innovations to facilitate further improvement in the provision of care.

Rwanda has taken several steps towards reducing the mother-to-child transmission of HIV through the establishment of their comprehensive national HIV programme. Currently, 97% of health facilities offer PMTCT services [19]. As of December 2013, 1.3% of women attending clinics for PMTCT services were HIV-positive, a substantial decrease from 10.8% HIV positivity among pregnant women in 2003 [19]. Further, the number of new infections among children decreased from 6400 in 2001, to less than 1000 in 2012 [18]. Still, levels of retention to the PMTCT cascade in Rwanda as well as individual- and facility-level factors associated with retention are not well understood.

This analysis utilized data collected from five health facilities in Rwanda to assess levels of retention and factors associated with retention to PMTCT programmes among mothers and infants.

## Methods

### Study design

We undertook a retrospective cohort study among pairs of HIV-positive pregnant women and their HIV-exposed infants (HEI) accessing PMTCT services in Rwanda. Specifically, this study aimed to investigate levels of retention among mother-infant pairs at various stages of the PMTCT cascade and assess individual and health facility characteristics that may influence retention in PMTCT programmes. At the time of the study, Rwanda had implemented Option B PMTCT guidelines (the provision of triple ART for all HIV-positive pregnant women during pregnancy up to the cessation of breastfeeding) for more than two years. Rwanda transitioned to Option B+, the provision of ART for all HIV-positive pregnant women for life, in April 2012, after the data collection period.

### Data collection

The retrospective review was conducted among HIV-positive women and infants registered in Option B antenatal and maternity services between November 2010 and February 2012. All PMTCT services were provided within the same unit of a particular facility. Facilities without maternity services referred women for delivery, but the women were expected to return for post-partum services and subsequent follow-up. Data were collected through a review of registers and medical files, as well as interviews performed with in-charges at the study health centres. Registers reviewed include the antenatal care (ANC), PMTCT, labour and delivery, HEI service, postnatal care (PNC), ART and early infant diagnoses registers. Data were extracted by trained data assistants and were directly entered into an MS ACCESS database. One to two informant interviews were conducted with the staff at each of the five study health facilities to collect data on health facility characteristics. Data were collected from March 2013 to May 2013.

In order to collect data on mothers and their infants from multiple registers, the data from these registers were linked. To obtain data on pregnant women, mothers newly diagnosed with HIV or with a known HIV-positive status were identified through the ANC registry by name and then their individual ANC identification numbers were noted. These unique identification numbers were then linked to the pre-ART registry patient numbers. The patient number was used to collect data from the ART, pharmacy and lab registers. To link the mother to the infant, data were also collected from the maternity, PNC and HEI registers. The mothers’ unique identification numbers are recorded in the infant's file, enabling the mother/infant pairing. “Unpaired” mothers were those for whom there was no linkage or information on infant follow up. To acquire infant characteristics, the HEI registers, HEI card and medical files (the polymerase chain reaction (PCR) and dried blood spot file results) were utilized. Infants with positive PCR results additionally had their details obtained from pre-ART, ART registers and patient files. No attempts were made to trace mothers across facilities.

Women who were referred for any reason were documented in the patient files, together with the dates of referral. These data were captured in the study database and the women subsequently removed from the denominator.

### Definition of variables

We defined retention as clinic attendance of the mother in addition to clinic attendance of the mother and infant as a pair. The time intervals along the PMTCT cascade used to assess retention were 30 days after entry into the PMTCT programme, at delivery, 6 weeks and 3, 6, 9 and 12 months post-delivery (postpartum time-points allowed a one-month interval on either side for data collection). Loss to follow-up (LFTU) was defined as missing three consecutive clinic visits.

Facility characteristics included facility location (urban or rural), staffing levels, vacancy rates, the number of ANC clients, volume of deliveries, number of HIV-positive pregnant women, number of doctor visits and onsite availability of CD4 testing.

### Site selection and sample size

Sites among those supported by Elizabeth Glaser Pediatric AIDS Foundation (EGPAF) were selected and stratified by type of facility and location for this study. Sites were included if they were an ART initiating site, had at least 40 HIV-positive pregnant women registered in ANC per year, had well-maintained patient records according to programme staff, and experienced minimal test-kit stock-outs. Utilizing this criterion, five health centres – two urban, three rural – were selected. We estimated a sample size of 474 pregnant women who were either newly diagnosed with HIV or had a known HIV-positive status. The sample size was based on an expectation of 50% (±4%) of the women attending the 12-month post-delivery visit.

### Endpoint derivation

To develop the time intervals along the PMTCT cascade, the time period from registration date to the end of the observation period was divided into six segments: 30 days after registration, delivery date, 6 weeks and 3, 6, 9 and 12 months post-delivery (postpartum time-points allowed a one month interval on either side for data collection). A mother/infant was considered retained at each time interval if there was at least one health centre visit or pharmacy pick-up at any time during that time interval. Retention at each time interval was measured dichotomously: a mother/infant received a “1” if there was at least one visit, or a “0” if there was none. For each mother-infant pair, retention was measured as a count outcome of how many visits out of a possible six visits were accomplished. Thus, the endpoint was the total number of visits (each with a value of one) accomplished out of the six expected.

To estimate retention 30 days after registration, the ART initiation date was utilized. For women who were already on ART, if their initiation date or the first ART refill date since their registration in PMTCT was either within 30 days or within two weeks of the 30 days after registration, respectively, the mother was considered retained. For retention at delivery (health facility or home), all available records were utilized that suggested that the delivery took place including place of delivery, delivery date (if available) and child date of birth (if available). If any one of these records was available, the mother was retained at delivery. With respect to retention post-delivery (6 weeks and 3, 6, 9 and 12 months), if there was any indication of receipt of ART at that time (±1 month), the mother was deemed retained. A similar process was used to estimate infant retention.

### Analysis

We estimated the proportion of mothers attending each study health centre visit by comparing the records of those found with the number that should have been seen at that particular visit. For mothers, at 30 days post-registration and at delivery, the denominator was the total number of registered mothers. For mothers at 6 weeks, and 3, 6, 9 and 12 months post-delivery, the denominator was the number of mothers retained at delivery. “Retention” was the proportion of the number of visits a mother attended to the total expected visits depending on gestational age at first visit. For infants, the initial denominator was the number of infants born alive, and then the number expected at each assessed time point, excluding known events such as death.

To assess factors associated with retention, we used generalized estimating equations (GEE) Poisson regression to calculate relative risks and the corresponding 95% confidence intervals (CIs) and *p*-values. We used GEE to account for the potential correlation of outcomes between women from the same health facility, assuming an exchangeable correlation structure. We also estimated robust standard errors, adjusting for the clustering within facilities. We included the following demographic, clinical and health facility variables in this analysis: age, parity, marital status, education, employment status, ART eligibility, CD4 count, urban or rural location of facility, facility deliveries, number of HIV-positive pregnant women per facility, HIV-trained nurses and number of doctor visits. Age, defined as a woman's age at registration or delivery, was estimated from the date of birth and the date of registration or delivery, and was scaled into 10 year increments in the analysis. Mother's parity was similarly retained as a continuous variable. Marital status was grouped into married/living as married versus not; education level – primary or less/secondary or more; and employment status – farmer, trader and other, including housewife. CD4 count was grouped into levels of 50 cells/mm^3^ and run as this grouped variable; ART eligibility was a dichotomous variable – ART for the mother's own health versus not (e.g. used solely for PMTCT). The health facility variables were based on annual (the preceding year's) data and were included in the analyses as continuous variables, while the total number of HIV-positive pregnant women was scaled into increments of 20 women.

In regression models, we analyzed the demographic, clinical and health facility factors associated with retention, with retention as a variable, the number of visits a mother made out of an expected number of six. This analysis utilized all the available data leading to a more robust estimate of retention. In the univariate (unadjusted) model, we included age in 10-year increments (years at registration), marital status (not married/married), education (primary or less/secondary or more), employment status (farmer, trader, other), parity (0–2, 3+), CD4 count categorized 50 mm^3^, known HIV status, ART eligibility, number of HIV-positive pregnant women (increments of 20 women) per facility, number of deliveries per facility, number of HIV-trained nurses, number of doctor visits and facility location (urban/rural). The number of doctor visits was omitted from the model because of collinearity. In the final multivariate (adjusted) model, we excluded education and the number of deliveries because they were not found to be significant in the univariate analysis.

Statistical analyses were conducted using Stata software (Version 12.1, StataCorp LP, College Station, Texas, USA).

### Ethical considerations

We obtained study approval from the Population Council Institutional Review Board and the Rwandan National Ethics Committee. Informed consent was waived, as the study was deemed minimal risk to study participants, the research could not practically be carried out if consent was required and the waiver of consent did not adversely affect the rights and welfare of the participants. All study personnel received training and certification in research ethics. Additionally, data assistants signed confidentiality agreements before interacting with the facility in-charges or working with data. All data were entered on password-protected computers into a password-protected database. When conducting the analyses, all personal identifiers were removed to ensure the confidentiality of the participants.

## Results

### Participant and health centre characteristics

**Figure 1:**
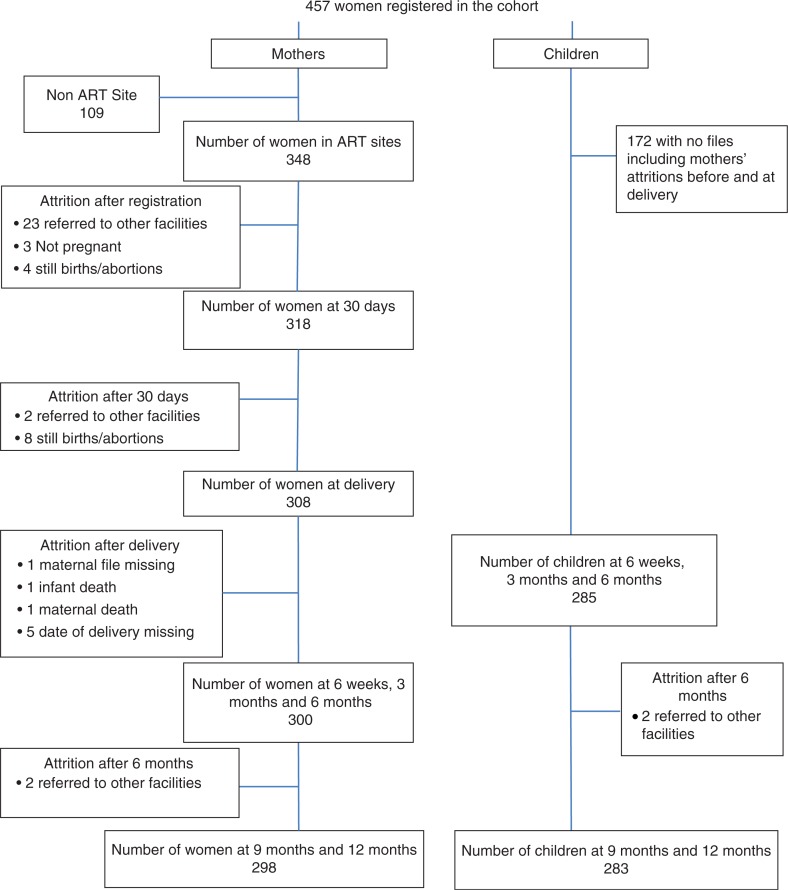
Mother and infant cascade.

A total of 457 women were planned to be followed in the study. Figure 1 presents a cascade of the mothers and infants at the various time intervals. One site with 109 records was excluded from the retention analysis after it was discovered that it was not an ART provision site at the time of the study period, which lowered the number of eligible women to 348. Consequently, although we present the descriptive characteristics of mothers and the health facilities for the original five facilities, only the 348 women, from four facilities, were included for the retention analyses. For the 457 infants born to these women, 172 files were not found, leaving an eligible sample of 285.

Table 1 presents the demographic and clinical characteristics of the paired and unpaired mothers, in addition to the services they received. Paired mothers were the women with located files who were able to be linked to their infants. Unpaired mothers were women who were unable to be linked to their infants. The mean ages and parities of paired and unpaired mothers were similar (mean age: 28.9 vs. 27.9 years, parity: 2.7 vs. 2.5). Although about 80% of women overall were married or living together, a greater proportion of unpaired compared to paired mothers were unmarried/living together, (17.1% vs. 10.2%; *p*=0.035). Overall, most (74%) women had primary education, but compared to paired mothers, a greater proportion of unpaired mothers recorded secondary education, (16.7% vs. 9.3%, *p*=0.024). More than half of the women were farmers (52.9%), though this was 63.3% of the paired mothers compared to 36.6% of the unpaired mothers, *p*<0.001.

**Table 1:** Descriptive characteristics of mothers participating in PMTCT programmes in five health centres in Rwanda

	Paired mothers		Unpaired mothers		Total	
			
	*N*	Mean (SD)	*N*	Mean (SD)	*N*	Mean (SD)
Age	283	28.9 (6.2)	172	27.9 (6.5)	455	28.5 (6.3)
Parity	276	2.7 (1.7)	167	2.5 (1.8)	443	2.6 (1.8)
CD4 count^a^	229	511.6 (223.8)	124	520.5 (223.9)	353	514.7 (223.6)
	
	***N***	**%**	***N***	**%**	***N***	**%**
	
Marital status						
Divorced/separated	15	5.4	7	4.2	22	4.9
Living as married	110	38.9	82	48.3	192	42.4
Married	144	50.9	59	34.8	203	44.9
Single	14	5.0	22	13.0	36	8.0
Total	283	100.0	170	100.0	453	100.0
Education						
None	38	14.7	22	13.6	60	14.3
Primary	197	76.1	113	69.8	310	73.7
Secondary	24	9.3	27	16.7	51	12.2
Total	259	100	162	100	421	100.0
Employment status						
Farmer	162	63.3	60	36.6	222	52.9
Housewife	57	22.3	66	40.3	123	29.3
Other	5	2	17	10.4	22	5.3
Trader	32	12.5	21	12.9	53	12.7
Total	256	100	164	100	420	100.0
Known HIV status						
Yes	173	60.7	99	57.6	272	59.5
No	112	39.3	73	42.4	185	40.5
Total	285	100	172	100	457	100
ART eligibility*						
Yes	187	73.6	83	64.3	270	70.5
No	67	26.4	46	35.7	113	29.5
Total	254	100	129	100	383	100
Number of ANC visits						
1	40	14.8	35	35.8	75	20.4
2	77	28.5	24	24.5	101	27.4
3	99	36.6	26	26.6	125	33.9
4+	55	20.3	13	13.3	68	18.5
Total	271	100.0	98	100.0	369	100.0
Type of regimen						
AZT/3TC/NVP	32	13.3	19	15.5	51	14.1
D4T/3TC/NVP	14	5.9	3	2.5	17	4.7
TDF/3TC/EFV	126	52.3	70	57.0	196	53.9
TDF/3TC/NVP	58	24.1	26	21.2	84	23.1
Others	11	4.6	5	4.1	16	4.4
Total	241	100.0	123	100.0	364	100.0

3TC=lamivudine; ABC=abacavir; ANC=antenatal care; ART=antiretroviral therapy; AZT=idovudine; D4T=stavudine; DNA PCR=deoxyribonucleic acid polymerase chain reaction; EFV=efavirenz; HIV=human immunodeficiency virus; NVP=nevirapine; PMTCT=prevention of mother-to-child transmission; SD=standard deviation

a CD4 at registration in PMTCT, (first ANC)

* *p*=0.059.

Only 18.4% of the women made the recommended four or more ANC visits; proportionately more unpaired (35.8%) compared to paired (14.8%) mothers had only one ANC visit, *p*<0.001. The most common treatment regimens among the women were tenofovir combination regimens (77%), while an additional 14% were on AZT combination regimens, with similar proportions among the paired and unpaired mothers.

### Description of health facilities

Five health centres were selected (three urban and two rural centres) for sampling. Using data collected in 2011, the average number of ANC clients among the five centres was approximately 1499, ranging from 880 to 2254 patients per centre (Table 2). There were roughly 70 HIV-positive pregnant women at each centre. In addition, an average of 647 deliveries took place at each facility. There was an average of approximately 29 health staffers and 10 HIV-trained nurses at each facility. Doctors visited the health centres approximately seven times each in 2011. Overall, the rural facilities had more staff per patient than the urban facilities.

**Table 2:** Description of the health facilities (*N*=5)

Variable^a^	Urban (Monthly Min–Max)	Urban (Monthly Min–Max)	Urban (Monthly Min–Max)	Rural (Monthly Min–Max)	Rural (Monthly Min–Max)	Mean (SD)
Number of ANC clients	880 (39–102)	1885 (121–192)	2254 (154–219)	1124 (72–119)	1350 (79–149)	1498.6 (562.4)
Number of HIV+ pregnant women	50 (2–9)	156 (7–19)	81 (3–9)	34 (2–7)	31 (1–5)	70.4 (51.8)
Number of deliveries	403 (19–63)	393 (54–93)	713 (65–130)	640 (38–74)	1086 (54–131)	647.0 (283.4)
Number of health staff	31	35	38	24	18	29.2 (8.2)
Number of HIV- trained nurses	11	10	8	12	10	10.2 (1.5)
Number of doctor visits^b^ last quarter	–	9	0	12	8	7.3 (5.1)

a Data for 2011

b doctors normally visit health facilities for consultation, supervision and support purposes.

### Programme retention

Table 3 shows the retention among women and infants across the PMTCT cascade with 95% CIs. Among all the women 58% (95% CI: 52%, 64%) were retained 12 months post-delivery, while the infants’ 12-month retention was 81% (76%, 86%). Retention for mothers paired with their infant (i.e. infant's file attached to mother's file) was 74% (68%, 80%), similar to the retention for their paired infant of 79% (73%, 85%) at 12 months. Analysis of retention of women without linked infant files shows a lower retention compared to the paired mothers; only 5% of unpaired women delivered at the clinic site, and about 30% were retained over the subsequent time periods. Overall, the majority of loss to retention was observed in the 30 days after antenatal registration. During this period, 33% of mothers overall and 25% of paired mothers were lost-to-follow-up. Losses tended to be minimal at the other time points.

**Table 3:** Mothers’^a^ and infants’ retention at specified time intervals

Section A						

	Mothers’ retention^a^			Infants’ retention		
		
Time Intervals	*N* expt.	*N* (Prop.)	95% CI	*N* expt.	*N* (Prop.)	95% CI
30 days	318	213 (0.67)	0.62, 0.72	N/A	N/A	N/A
Delivery	308	205 (0.67)	0.62, 0.72	N/A	N/A	N/A
6 weeks	300	203 (0.68)	0.63, 0.73	285	218 (0.77)	0.71, 0.81
2–4 months	300	201 (0.67)	0.62, 0.72	285	243 (0.85)	0.81, 0.89
5–7 months	300	193 (0.64)	0.59, 0.69	285	259 (0.91)	0.88, 0.94
8–10 months	298	185 (0.62)	0.56, 0.68	283	247 (0.87)	0.83, 0.91
11–13 months	298	172 (0.58)	0.52, 0.64	283	228 (0.81)	0.76, 0.86
**Section B**						

	**Paired mother's retention** **b**			**Paired infants’ retention**		
		
**Time Intervals**	***N*****expt**.	***N*** **(Prop.)**	**95% CI**	***N*****expt**.	***N*** **(Prop.)**	**95% CI**

30 days	200	147 (0.74)	0.68, 80.0	N/A	N/A	N/A
Delivery	200	200 (0.100)	N/A	N/A	N/A	N/A
6 weeks	198	166 (0.84)	0.79, 0.89	198	161 (0.82)	0.76, 0.86
2–4 months	198	167 (0.84)	0.79, 0.89	198	173 (0.88)	0.82, 0.92
5–7 months	198	165 (0.83)	0.78, 0.88	198	177 (0.90)	0.85, 0.93
8–10 months	196	155 (0.79)	0.73, 0.85	196	168 (0.86)	0.81, 0.91
11–13 months	196	144 (0.74)	0.68, 0.80	196	154 (0.79)	0.73, 0.85
**Section C**						
			
	**Non-paired mother's retention** **a**					
				
**Time intervals**	***N*** **expt**.	***N*** **(Prop.)**	**95% CI**			
			
30 days	118	66 (0.56)	0.47, 0.65			
Delivery	108	5 (0.05)	0.009, 0.09			
6 weeks	102	37 (0.37)	0.28, 0.46			
2–4 months	102	34 (0.34)	0.25, 0.43			
5–7 months	102	28 (0.28)	0.19, 9.37			
8–10 months	102	30 (0.30)	0.21, 0.39			
11–13 months	102	28 (0.28)	0.19, 0.37			

a Analysis based on 348 mothers

b paired mothers; linked mother-infant records.

### Factors associated with retention

Table 4 presents retention at specified time intervals by selected demographic and clinical factors. Single mothers had the lowest retention at delivery (48%; 95% CI: 30%, 66%), 9 (41%; CI: 23%, 59%) and 12 months (41%; CI: 23%, 59%). Divorced/separated mothers had the lowest retention at six weeks (43%; CI: 17%, 69%), three (36%; CI: 11%, 61%) and six months (36%; CI: 11%, 61%). Retention across the time periods was similar by education levels, while women whose occupation was listed as “other” (than farmer, housewife or trader), had the lowest retention across all the time periods from delivery compared to other occupations: 35% at delivery, 53% at 6 weeks and 3, 6 and 9 months, and 47% at 12 months. There was little difference in the proportions retained between mothers with known HIV status and those newly diagnosed across the specified time intervals, with 59% (CI: 52%, 66%) of the mothers of known status retained at 12 months, against 56% (CI: 47%, 65%) of newly diagnosed mothers. Mothers eligible for ART for their own health were better retained across all the time periods, compared with those not eligible and receiving ART solely for PMTCT, 66% (CI: 59%, 73%), were retained at 12 months versus 47% (CI: 37%, 57%), respectively, *p*<0.001. There was little difference in retention between mothers who attended the recommended four or more ANC visits, versus those who attended three or fewer, 66% (CI: 51%, 80%), versus 65% (CI: 53%, 78%) at 12 months.

**Table 4:** Retention among mothers in PMTCT programmes at specified time intervals by demographic characteristics

	30 days		Delivery		6 weeks		3 months		6 months		9 months		12 months	
							
	Prop	95% CI	Prop	95% CI	Prop	95% CI	Prop	95% CI	Prop	95% CI	Prop	95% CI	Prop	95% CI
Marital status														
Divorced/separated	0.80	0.60, 0.100	0.80	0.60, 0.100	0.43	0.17, 0 69	0.36	0.11, 0.61	0.36	0.11, 0.61	0.50	0.24, 0.76	0.57	0.31, 0.83
Living as married	0.66	0.59, 0.73	0.64	0.57, 0.71	0.66	0.59, 0.73	0.66	0.59, 0.73	0.64	0.57, 0.71	0.61	0.54, 0.68	0.57	0.49, 0.64
Married	0.67	0.57, 0.77	0.75	0.66, 0.84	0.79	0.70, 0.88	0.78	0.69, 0.87	0.75	0.69, 0.87	0.74	0.65, 0.83	0.66	0.56, 0.76
Single	0.64	0.51, 0.83	0.48	0.30, 0.66	0.52	0.34, 0.70	0.52	0.34, 0.70	0.45	0.34, 0.70	0.41	0.23, 0.59	0.41	0.23, 0.59
Education														
None	0.71	0.57, 0.85	0.71	0.57, 0.85	0.69	0.51, 0.82	0.60	0.44, 0.76	0.60	0.44, 0,76	0.57	0.41, 0.73	0.51	0.34, 0.68
Primary	0.65	0.60, 0.72	0.64	0.58, 0.70	0.66	0.59, 0.72	0.66	0.59, 0.72	0.63	0.56, 0.69	0.62	0.55, 0.69	0.58	0.51, 0.65
Secondary	0.67	0.52, 0.82	0.61	0.45, 0.76	0.61	0.45, 0.77	0.61	0.45, 0.77	0.58	0.42, 0.74	0.53	0.37, 0.69	0.53	0.37, 0.69
Occupation														
Farmer	0.62	0.53, 0.71	0.82	0.75, 0.89	0.72	0.63, 0.81	0.70	0.61, 0.79	0.69	0.60, 0.78	0.66	0.57, 0.75	0.59	0.49, 0.69
Housewife	0.67	0.58, 0.76	0.50	0.41, 0.59	0.61	0.52, 0.70	0.63	0.54, 0.72	0.57	0.48, 0.66	0.56	0.47, 0.65	0.55	0.47, 0.65
Other	0.71	0.49, 0.93	0.35	0.12, 0.58	0.53	0.29, 0.77	0.53	0.29, 0.77	0.53	0.29, 0.77	0.53	0.29, 0.77	0.47	0.23, 0.71
Trader	0.69	0.56, 0.82	0.67	0.54, 0.80	0.65	0.52, 0.78	0.60	0.46, 0.74	0.58	0.42, 0.70	0.56	0.42, 0.70	0.54	0.40, 0.68
Known HIV status														
Yes	0.66	0.60, 0.74	0.70	0.63, 0.77	0.69	0.62, 0.76	0.68	0.61, 0.75	0.67	0.60, 0.74	0.65	0.58, 0.72	0.59	0.52, 0.66
No	0.68	0.59, 0.76	0.61	0.52, 0.70	0.66	0.57, 0.75	0.66	0.57, 0.75	0.60	0.51, 0.69	0.58	0.49, 0.67	0.56	0.47, 0.65
Eligible for ART														
Yes	0.72	0.66, 0.78	0.72	0.66, 0.78	0.76	0.70, 0.82	0.77	0.71, 0.83	0.73	0.69, 0.79	0.71	0.65, 0.77	0.66	0.59, 0.73
No	0.66	0.56, 0.76	0.63	0.53, 0.73	0.60	0.50, 0.70	0.55	0.45, 0.65	0.54	0.44, 0.64	0.52	0.42, 0.63	0.47	0.37, 0.57
Number of ANC visit														
1–3	0.74	0.64, 0.86	0.82	0.73, 0.91	0.74	0.63, 0.85	0.77	0.65, 0.87	0.72	0.61, 0.84	0.70	0.58, 0.82	0.65	0.53, 0.78
4+	0.65	0.50, 0.80	0.80	0.69, 0.93	0.85	0.74, 0.96	0.80	0.68, 0.92	0.80	0.68, 0.92	0.68	0.54, 0.82	0.66	0.51, 0.80

ANC=antenatal care; ART=antiretroviral therapy; CI=confidence interval; HIV=human immunodeficiency virus; PMTCT=prevention of mother-to-child transmission; Prop=proportion retained.

In the final model, employment (defined as farmer versus non-farmer) was excluded as it failed to reach significance in an earlier multivariate model. The number of HIV-trained nurses was also dropped as the model did not converge. Table 5 presents the univariate and final multivariate models. In the final model, mothers who were married/living as married were 1.26 (95% CI: 1.11, 1.43) times more likely to be retained than mothers who were single or divorced/ separated. Mothers who were eligible for ART for their own health were 1.39 (95% CI: 1.12, 1.73) times more likely to be retained than those who were receiving ART solely for PMTCT. CD4 count was independently and positively associated with retention (ARR: 1.02 per 50 mm^3^ increase in CD4; 95% CI: 1.01, 1.03). Mother's parity and age, HIV status at the time of registration, number of HIV-positive pregnant women seen at a facility and facility location (urban/rural) were not significantly associated with retention.

**Table 5:** Regression model of factors associated with retention among women, unadjusted and adjusted relative risks, (*n*=348)

	Unadjusted relative risk			Adjusted relative risk^a^		
		
Variable	RR	95% CI	*p*	RR	95% CI	*p*
Married	1.26	1.08, 1.47	0.003	1.26	1.11, 1.43	<0.001
Age/10 years	1.09	1.00, 1.19	0.042	1.01	0.93, 1.03	0.772
Education						
Primary or less	1.02	0.87, 1.21	0.76	Excluded from final model		
Secondary or more		0.78, 1.20	0.75			
Parity	1.12	1.01, 1.24	0.026	1.02	0.89, 1,17	0.752
HIV status: Known	1.07	0.96, 1.18	0.222	0.98	0.92, 1.04	0.455
ART eligible: Yes	1.27	1.14, 1.42	<0.001	1.39	1.12, 1.73	0.003
CD4/50 mm^3^	0.99	0.98, 1.01	0.308	1.02	1.01, 1.03	<0.001
Employment						
Farmer	1.19	1.05, 1.33	<0.001	Excluded from final model		
Trader	1.05	0.89, 1.24	0.552			
Other	0.89	0.68, 1.16	0.394			
Facility location: Urban	0.79	0.74, 0.87	<0.001	0.39	0.12, 1.32	0.133
Number of HIV-positive pregnant women/20	0.96	0.95, 0.98	<0.001	1.14	0.91, 1.44	0.251
Number of facility deliveries	0.99	0.99, 1.00	0.406	Excluded from final model		
Number of HIV- trained nurses	1.20	1.14, 1.28	<0.001			

RR=relative risk

a adjusted for all covariates listed.

## Discussion

We found that the majority of loss-to-follow-up for mothers occurred early in the course of the PMTCT programme, with loss of 33% of mothers overall by 30 days after registration into the programme (26% for paired and 44% for unpaired mothers). The high early loss-to-follow-up in our study is similar to that in a Malawi study in which the highest loss of pregnant HIV-positive women occurred soon after diagnosis [15]. After this initial loss, retention decreased by only 10% between 6 weeks and 12 months post-delivery. Retention for mothers was 68% at six weeks post-delivery, decreasing to 58% by 12 months post-delivery.

Overall retention for infants was better than for mothers, with 81% retained at 12 months. For mothers and infants whose records were able to be linked as mother-baby pairs, retention was similar for mothers and infants, 74% at 12 months for mothers and 79% for infants. Factors associated with maternal retention were marital status, parity, CD4 count (per 50 mm^3^ increase), whether ART was being received for maternal health, rural health facility location and number of HIV-positive pregnant women seen at the facility.

The proportion of women evaluated as mother-infant pairs retained at 12 months post-delivery found in this study (74%) is similar to data reported by the Rwandan Ministry of Health, which indicated that 70% of women are retained [20]. The 81% infant retention at 12 months is significantly higher than the retention rates for Sub-Saharan Africa reported in systematic review and met-analysis [21]. LFTU among HEI at three months had a pooled estimate of 34% from 11 studies in African countries. A South African study reported a LTFU of 85% at 12 months. This study and most of the other studies were on programmes that offered single-dose nevirapine. These programmes had higher LTFU compared to programmes that offered more intensive regimens such as Option B/B+ [21].

The non-paired mothers appeared to have delivered elsewhere, as only 5% were found in the delivery records. Women may deliver in facilities other than where they have registered, as they may perceive the quality of care to be better in these facilities, or they may deliver at home, where family members can provide support. Many of the unpaired mothers (107/172) were from a single urban facility. During the study period, this facility had begun offering maternity services, so it was observed for a limited time (seven months). Compared to the paired mothers, unpaired mothers tended to be younger, be unmarried, have more secondary education and have occupations other than farming. They also attended ANC less frequently. Literature has found a positive association between levels of education and better retention [11,15]; in our study, this inverse relationship observed in the bivariate analysis could be related to location as more unpaired mothers were found in urban clinics, where retention appeared to be lower.

However, while women attending urban facilities appeared to be less likely to be retained compared to women attending facilities in rural areas, this was not significant in the adjusted model. This finding is at variance with the tendency of higher LFTU of pregnant and postpartum HIV-positive women seen in large urban clinics reported by Tethani in Malawi [15]. Strategies should be developed to better retain single, HIV-positive pregnant women. Such strategies could include peer support groups linked to income-generating or skills-building activities, tracing with active follow-up using mobile phone texting/voice and the use of social media and community health workers. Peer support group approaches have proved to be beneficial in improving retention in some settings [22,23], as has the use of mobile phone texting [24]. In addition, the use of conditional cash transfers could be explored.

ART eligibility was positively associated with retention. This finding is consistent with findings that pregnant women who have needed ART for their own health have better retention compared to women who have not and are receiving ART solely for PMTCT [15]. These women may better understand the need to consistently return to the health facility because they have experienced symptoms. By comparison, women who are not ART eligible may not perceive themselves to be ill. The positive association of CD4 count and retention may be a function of improved CD4 status as a result of medication, but may be also a result of referrals to better care for symptomatic women. Positive associations between CD4 count and retention were reported also among patients on ART in Tanzania, Uganda and Zambia [25]. As an independent predicator of retention, parity may reflect the profile of women attending rural facilities, who tend to be older and married.

The strengths of this analysis include the utilization of numerous registers, the statistical methods used to assess retention and the novel exploration of health facility factors and their impact on retention in Rwanda. A limitation of this study was the limited number of health facilities, though these health facilities were likely to be similar in operation to other health facilities in Rwanda. Another limitation is that retention may have been overestimated, as the availability of good records was a criterion of site selection. The number of participants was smaller than the expected sample size, which reduced the power to detect differences in retention by the study variables. Finally, the data collection methods were comprehensive, but complicated because of the number of paper-based registers, and the lack of an electronic linkage mechanism among the registers and between mother and infant records.

## Conclusions

Our data suggest that unmarried, apparently healthy, HIV-positive pregnant women are at the greatest risk of being lost-to-follow-up and may require additional support for programme retention. With new recommendations that lifelong ART should be started in all HIV-positive individuals, including pregnant women, strategies are needed to identify, provide support and trace those women at highest risk of LFTU. These strategies could include risk profiling and support groups for these women. Our study provides further evidence that a targeted supportive approach, allowing a focus of additional resources to the group of women at the highest risk of being lost, would be appropriate. Areas for further research include closer examination of the factors that could improve retention in urban areas, such as the use of mobile phones/texting and social media, peer groups linked to income generating/skills building activities. Research on facility-level factors could include enhanced counselling, and the optimization of patient tracking, staffing levels and configuration.
